# Unwanted cardiopulmonary resuscitation against patients’ “Do Not Attempt Resuscitation” orders in community settings in Japan: A narrative review

**DOI:** 10.1111/ggi.14993

**Published:** 2024-10-01

**Authors:** Kaku Kuroda, Kaori Ito, Takeshi Uemura

**Affiliations:** ^1^ Division of Geriatrics and Aging, Department of Medicine University of Rochester Rochester New York USA; ^2^ Department of General Internal Medicine University of Toyama Toyama Japan; ^3^ Department of Surgery, Division of Acute Care Surgery Teikyo University School of Medicine Tokyo Japan; ^4^ Brookdale Department of Geriatrics and Palliative Medicine Icahn School of Medicine at Mount Sinai New York New York USA

**Keywords:** cardiopulmonary resuscitation, Do Not Resuscitate order, Japan

## Abstract

We aimed to synthesize existing research to elucidate the underlying factors and causes responsible for the high prevalence of unwanted cardiopulmonary resuscitation (CPR) occurring outside a hospital setting in Japan despite patients' Do Not Attempt Resuscitation (DNAR) orders. We conducted a narrative review by searching PubMed, EMBASE, and Scopus for English literature, and Google Scholar for Japanese literature. The key factors we identified included lack of documentation of resuscitation preferences, variation in the perception of other life‐sustaining measures associated with DNAR, non‐inclusion of the patient in discussions of goals of care, unlegislated and unstandardized DNAR orders, emergency medical service activation by the family or facility, the Fire Service Act that mandates life‐saving measures irrespective of the presence of advance directives, fire department protocols and CPR decision‐making, and death pronouncement authorization limited to physicians. This study identified the multifaceted factors and the potential triggers for unwanted CPR despite DNAR orders. These findings underscore the urgent need for comprehensive interventions encompassing educational initiatives, ethical considerations, systemic reforms, and legal adjustments to prevent future unwanted CPRs in Japan. **Geriatr Gerontol Int 2024; 24: 1093–1098**.

## Graphical abstract

We identified the key factors contributing to unwanted cardiopulmonary resuscitation despite Do Not Attempt Resuscitation (DNAR) orders in Japan. These were related to unstandardized DNAR orders, lack of documentation of resuscitation preferences, variation in the perception of DNAR orders, goals of care discussion, and legal climate, including the Fire Service Act and death pronouncement authorization.

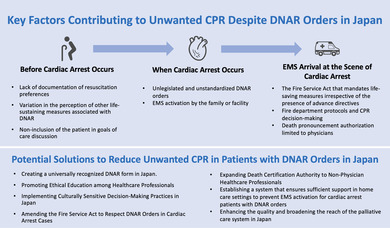

## Introduction

Japan precedes the rest of the world in population aging, which could lead to a surge in deaths. As of January 2024, the size of the population aged 65 and older was estimated to be 36 million, which was 29.1% of the total population.^1^ This population percentage of older adults is the highest in the world, followed only by Italy at around 23%.^2^ This notably large aged population has led to a rapidly growing number of deaths in Japan. In 2023, 1.57 million individuals died,^3^ and the number is projected to exceed 1.6 million in 2030.^4^ Given this dire situation, advance care planning (ACP) has become more important because it enables older adults to articulate and convey their treatment preferences when they are still cognitively intact.

With this background, the “Do Not Attempt Resuscitation” (DNAR) order, which is more commonly known as “Do Not Resuscitate” (DNR) in countries other than Japan, has played an essential role as an instrumental mechanism for aligning medical interventions with the patient's preferences and values.^5^ The DNAR order safeguards patients’ autonomy and preserves their dignity amidst end‐of‐life scenarios.^6^ However, there are a substantial number of cases reported in which unwanted cardiopulmonary resuscitation (CPR) was administered in Japan despite the presence of a DNAR order. In a cross‐sectional study concerning out‐of‐hospital cardiac arrests in Sagamihara city, 95.6% (43 out of 45) of patients who had DNAR orders received CPR.^7^ Another study similarly reported that 98% (120 out of 122) of patients with DNAR orders who had an out‐of‐hospital cardiac arrest received CPR in Okayama city.^8^ Additionally, in a separate cross‐sectional study of 43 frail individuals with cardiac arrest, 16 had DNAR orders, of whom 13 (81.3%) received CPR.^9^ This disturbing discrepancy between patients' desired care in the end‐of‐life period and the actual medical interventions they receive raises pressing questions about the factors and circumstances that contribute to unwanted CPR.

Although research on unwanted CPR in Japan exists, to our knowledge, no systematic or narrative reviews with in‐depth investigations of unwanted CPR in the Japanese healthcare context are available in English.

Thus, we aimed to combine existing research to uncover the underlying factors and reasons that lead to the occurrence of unwanted CPR contrary to patients' DNAR orders outside a hospital setting in Japan.

## Methods

We conducted literature searches using PubMed, EMBASE, and Scopus to identify articles written in English, and Google Scholar searches to locate articles written in Japanese. The search terms and strategy are outlined in Tables S1. and S2.

## Results

We identified the following factors as contributing to unwanted CPR despite DNAR orders. The identified factors have been chronologically organized based on what would happen before/when/after cardiac arrests occur. We have consolidated the pivotal findings of these articles in Table S1.

### Factors leading to unwanted CPR despite DNAR orders

#### Before cardiac arrest occurs

- *Lack of Documentation of Resuscitation Preferences*: Several studies showed incidences of confirming DNAR orders verbally and the lack of documentation of DNAR orders. In a study of 124 patients admitted to a teaching hospital in 1989 or 1999, none had a signed DNAR order, despite 113 patients' families confirming verbal DNAR orders.^10^ The study indicated a consistent lack of DNAR documentation over the 10 years from 1989 to 1999.^10^ Another cross‐sectional study of data between 2014 and 2018 reported that only 15 out of 270 cases (5.6%) with known DNAR orders carried the documentation of DNAR orders at the time of cardiac arrest.^11^ In another survey study, of 243 emergency medical technicians (EMTs) who had experienced transporting out‐of‐hospital cardiac arrest patients with DNAR orders between 2015 and 2019, only 9 (3.7%) confirmed patients' written DNAR orders.^8^ In another cross‐sectional study, among 45 patients who had DNAR orders between 2019 and 2020, 12 (26.7%) had documentation to confirm their DNAR orders.^7^, ^10^
- *Variation in the Perception of Other Life‐sustaining Measures Associated with DNAR Orders*: Existing literature indicates that a variation in the comprehension of DNAR orders might be contributing to the inappropriate execution of these orders. In a survey study conducted in 2016 that administered three hypothetical scenarios to residents (56%) and attending physicians (44%), 22–33% answered that they would withhold life‐sustaining measures other than CPR when a patient had a DNR order.^12^ Furthermore, 16% to 26 % answered that they would perform electric shock on patients with DNR orders even though they would stop other non‐CPR measures, which reflects a serious misapplication of DNAR orders among Japanese physicians. Another survey study of 384 nurses conducted in 2017 found that they would withhold extracorporeal membrane oxygenation more likely than chest compressions when patients have DNAR orders.^13^ Similarly, in survey of 595 physician members of the Japanese Society of Intensive Care Medicine in 2017, a significant percentage of responders recommended withholding non‐CPR procedures, such as hemodialysis (79.3%) in patients with DNAR orders.^14^ Furthermore, in a survey study of the ethics committee chairs of hospitals across Japan conducted in 2020, 104 out of 188 hospitals (55%) indicated that they had had cases where non‐CPR medical procedures were withheld or withdrawn because of DNAR orders.^15^
- *Non‐inclusion of the Patient in Goals of Care Discussion*: Numerous articles have revealed a concerning trend of limited patient engagement in discussions on DNAR decisions. In a retrospective cohort study, only about 6% of the total of 447 patients (300 with cancer and 147 without cancer) had participated in discussions about their preferences on life‐sustaining measures with their physicians, which were instead occurring between the patients’ spouses or children and physicians.^16^ In the same study, even among 185 cancer and 57 non‐cancer patients without cognitive impairment, only 14 (7.6%) and 6 (10.5%) patients, respectively, engaged in their decisions on having DNR orders.^16^ In a different cross‐sectional study conducted in a palliative care unit, among 163 patients with decisional capacity, 28% were not involved in discussions regarding having DNR orders.^17^ While some studies did not mention patients' medical decision‐making capacity, many patients had not engaged in discussion about their preferences on life‐sustaining measures, and family or physicians generally made the decisions.^18^, ^19^, ^20^ This low participation rate might also stem from healthcare professionals' limited perspectives on the importance of patients' involvement in discussions about their preferences for life‐sustaining measures. A survey of 595 physicians found that 325 physicians (60.2%) never discussed DNAR orders with the patient present.^14^ In a similar survey of 384 nurses, only 16 nurses (6.5%) consistently engaged patients in DNAR decision‐making, while 104 (42.1%) only occasionally involved patients in these decisions.^13^

#### When cardiac arrest occurs

- *Unlegislated and Unstandardized DNAR Orders in Japan*: There is a lack of consensus regarding the proper procedure for communicating the DNAR order status between patients, their families, and healthcare professionals. A cross‐sectional study published in 2017 noted that the absence of standardized and shareable formats of DNAR orders at the community level could lead to confusion about patients' DNAR orders during ambulance calls.^18^ A nationwide survey directed at ethics committees of 195 hospitals conducted in 2020 uncovered that 111 hospitals (57%) did not possess any formats for DNAR orders.^15^ In the same study, among the other 84 hospitals that had a DNAR format, 82 (96%) exclusively utilized their own formats that were not shareable with other hospitals, facilities, and emergency medical services (EMS).^15^
- *EMS Activation by Family or Facility*: The Fire and Disaster Management Agency of the Ministry of Internal Affairs and Communications reported that in 2017, there were 2015 ambulance calls for a cardiac arrest where the requester of the ambulance refused CPR owing to the patient's DNAR orders when EMTs arrived at the scene.^21^ Questionnaires administered to EMTs in 2017 indicated that among 63 EMTs who had transported out‐of‐hospital cardiac arrest patients with terminal cancer who had DNR orders to hospitals, 60 (95.2%) had experienced being informed by family members of the DNAR order's presence while CPR was underway.^22^
  This tendency to activate EMS when cardiac arrests occur in patients with a DNAR order has also been reported in nursing homes. In a separate study focusing on 270 cardiac arrest cases with DNAR orders where EMS were activated between 2014 and 2018, 105 cases (38.9%) were from nursing homes.^11^ Another investigation into the utilization of DNAR orders within nursing homes in 2012 reported that out of 108 surveyed facilities, 43.6% indicated that they would request an ambulance for all cardiac arrest cases, irrespective of DNAR orders.^23^ Another qualitative study conducted in 2015 identified various factors regarding why an ambulance was called for cardiac arrests in terminal cancer patients with wishes to have DNAR orders. These encompassed an unclear legal climate surrounding DNAR orders, a lack of medical support and end‐of‐life care systems in both home and nursing home settings, and inadequate knowledge, recognition, and preparedness among family members.^24^

#### EMS arrival at the scene of cardiac arrest

- *The Fire Service Act*: The Fire Service Act is a national law that was originally issued in 1948. The Fire Service Act governs how EMS should carry out emergency services. It mandates EMS to initiate CPR and transport the patient to an emergency department of the nearest hospital available when an ambulance is summoned for out‐of‐hospital cardiac arrest.^25^ This Act does not mention what to do when the patient has a DNAR order; thus, EMS often feel compelled to perform CPR, irrespective of DNAR orders. Among 63 surveyed EMTs in 2017, 97% reported that they had had cases where they performed CPR for terminal cancer patients with DNAR orders to comply with the Fire Service Act.^22^ In another survey study published in 2022 involving 243 EMTs involved in out‐of‐hospital cardiac arrest cases with DNAR orders, 155 (65%) acknowledged that they had employed a “slow code” approach in the past to comply with the law.^8^
- *Fire Department Protocols and CPR Decision‐Making*: Protocols established by fire departments in various areas are aligned with the Fire Service Act. Consequently, these protocols lack provisions for situations where CPR should be avoided. For instance, the protocol designed by the Tokyo Fire and Disaster Management Agency and Saitama Fire Department specifies that CPR should be initiated immediately in all cases and only be terminated following consultation with the primary care physician (PCP).^26^, ^27^, ^28^ In a study conducted in 2017, 38% of the surveyed 1079 EMTs expressed that they experienced difficulties in reaching the PCP while simultaneously conducting CPR to obtain the order to halt resuscitation.^29^ These fire department protocols significantly influence the decision to transport the patient to a hospital.
- *Death Pronouncement*: In Japan, death pronunciation and certification are exclusively performed by physicians, as mandated by law.^30^ Physicians are required to conduct a physical examination of the patient before pronouncing death. Consequently, cardiac arrest patients with DNAR orders are frequently transferred to hospitals because physicians are not readily available to pronounce death at the scene. In a survey study conducted in 2014, approximately 70% of physicians suggested that they would defer the decision on whether to stop CPR to the hospital emergency physicians because EMTs would not be able to pronounce death even after halting CPR in the physical absence of the physician.^18^ In one study using data between 2014 and 2018, of the total 270 cardiac arrest cases with DNAR orders, only 22 patients (8.1%) were pronounced dead at the site owing to the presence of a PCP, whereas 248 patients (91.9%) were transferred to medical institutions while receiving CPR.^11^

## Discussion

We identified the factors influencing the occurrences of unwanted CPR despite DNAR orders in Japan. The findings presented in this study shed light on an intricate landscape characterized by a myriad of the following interconnected factors that contribute to unwanted CPR.

### Lack of consensus on DNAR order format

The results reveal that the absence of legislated DNAR orders in Japan significantly impacts multiple phases of care, from before cardiac arrest to after it happens. Studies have indicated that many healthcare facilities either lack standardized DNAR formats or utilize internal documents not intended for sharing outside their facilities.^15^, ^18^ In comparison, in the United States, the utilization of the Physician Orders for Life‐Sustaining Treatment (POLST) form as a universal, state‐level document guides both EMS and healthcare professionals in adhering to patients' treatment preferences regarding life‐sustaining interventions.^31^ While there are variations in implementing POLST forms among different states,^32^ using POLST forms effectively prevents unwanted CPR. In contrast, although the Japanese Association of Clinical Ethics published a Japanese version of the POLST form in 2015,^33^ the Japanese Society of Intensive Care Medicine released statements discouraging its use in 2017. According to the statements, there was a concern that the POLST form may lead to inappropriate discouragement for life‐saving efforts,^34^ primarily because many physicians and nurses misunderstand DNAR orders and misuse them to withhold other life‐sustaining procedures.^13^, ^14^ Therefore, to create a universally shareable format for DNAR orders, improving healthcare providers’ understanding of the correct implication of DNAR orders through education aimed at all medical professionals and rebuilding the Japanese version of the POLST form seems crucial.

### Communication deficits regarding ACP

The literature analysis reveals various ethical concerns intertwined with the lack of patient involvement in ACP.^16^, ^17^, ^18^, ^19^, ^20^ When a family makes a decision, they may tend to regard withholding life‐sustaining procedures as abandonment or even killing, which strongly affects physicians' decision‐making.^35^ Decisions made jointly only by physicians and families, who can be influenced by a sense of guilt and fear, without the patient's involvement can result in aggressive life prolongation. Nonetheless, it is crucial to recognize that the cultural context may be playing a role in this tendency of not involving the patient in the decision‐making process in Japan. Japan's high‐context culture underscores implicit cues over explicit communication,^36^ and the preference for family‐oriented decision‐making rather than individual self‐determination creates a distinct cultural contrast to Western approaches.^36^, ^37^ Additionally, although there is no solid evidence, it has been hypothesized that both the low healthcare costs stemming from universal insurance and the reimbursement system of fee‐for‐service payments during acute phases in Japan might be contributing to the tendency of continuing aggressive life prolongation.^35^

Additionally, it is crucial to recognize the absence of ACP as a potential contributor to unwanted CPR. ACP is widely acknowledged for averting undesired aggressive treatments by aligning patients' values with the care provided.^38^ A multi‐country survey showed that Japanese people might have a greater tendency to choose DNR than those in the United States, Italy, and Brazil.^39^ Contrary to this tendency, as described in our results, the rate of documenting advance directives is low in Japan.^39^ Thus, many Japanese patients who would have chosen DNAR might remain full code owing to the lack of conversation and documentation, which means that the number of actual cases of “unwanted” CPR could be higher than reported. Also, because of a noticeable deficit in documentation related to advance directives in Japan, as indicated by our findings,^7^, ^8^, ^11^, ^40^ it is often unclear in an emergency whether patients' preferences on life‐sustaining measures have already been discussed.

### The necessity for ethical education among healthcare professionals

The findings demonstrate that a notable concern revolves around healthcare providers' comprehension and approach toward DNAR orders. Several articles mention a misunderstanding of the definition of DNAR orders among healthcare professionals.^12^, ^13^, ^14^, ^15^ Also, there is evident confusion between DNAR orders and comfort measures‐only orders.^41^ On top of this misunderstanding and confusion, there is a concern that providers tend not to discuss DNAR appropriately when the patient is against undertaking CPR. Furthermore, because of this concern, the Japanese Society of Intensive Care Medicine discouraged the Japanese version of the POLST form. Despite this concern, while various guidelines for end‐of‐life care have been issued by entities, none provide specific and clear recommendations on managing DNAR orders within the context of end‐of‐life care.^25^ These concerns regarding ethics among healthcare professionals underscore the necessity for comprehensive education to foster a clear understanding and effective implementation of DNAR orders.

### Legal climate and regulatory ambiguities

Several articles describe the situation where EMS have no choice but to comply with the law and perform CPR despite knowing that the patient has a DNAR order.^22^, ^25^ Japan's Fire Service Act contains no provisions or descriptions about DNAR orders. Additionally, there is a lack of standardized forms with legal validity for documenting DNAR orders. Consequently, within the current legal framework, the Fire Service Act mandates EMS to conduct CPR regardless of the presence of DNAR orders. The Act was originally issued in 1948, a time when the concept of DNAR did not exist in Japan. Since then, no major adjustments have been made. Updating the Act regarding appropriate responses in cardiac arrest cases with DNAR orders appears essential.

Another crucial aspect is the legal authorization for death pronouncement. The findings suggest that some patients have been transferred to hospitals solely in order to enable the declaration of death.^27^ Because only medical doctors can pronounce death under Japan's Medical Practitioner's Act, the capability to pronounce death at home remains limited. This limitation results in superfluous transfers to the emergency department and unwanted CPR. In contrast, in the United States, hospice‐registered nurses, physician assistants, and nurse practitioners can pronounce a patient's death, albeit with variations across different states.^42^ Expanding the authorization of certifying deaths to other appropriate healthcare professionals could increase the capability for death pronouncements at home, thus reducing unwanted CPR and transfer to hospitals solely for death pronouncement purposes.

### Challenges within the healthcare system

A recurring concern is the activation of EMS by family members and caregivers when an anticipated change in condition occurs in individuals receiving home medical care or nursing care. As stated above, EMS must conduct CPR, complying with the Fire Service Act, regardless of DNAR orders within the current legal climate. Therefore, EMS activation for patients with DNAR orders has the potential to initiate unwanted CPR inadvertently. This issue may arise owing to insufficient support for home care patients within the current healthcare system, leaving caregivers with no alternative to calling an ambulance. Home medical care is an essential resource for caregivers of patients with DNAR orders to prevent unnecessary ambulance calls. In Japan, physician‐led home medical care plays the role of home hospice. However, the number of home‐visiting physicians is currently insufficient to meet the demands of the growing elderly population who wish to spend their end‐of‐life period at home, and the need for home‐visiting physicians is anticipated to rise further.^43^ Moreover, over 70% of home‐visiting physicians were found to experience considerable strain due to the demanding nature of their 24‐h on‐call duty.^44^ Considering these facts, there is an urgent need to establish a system that guarantees sufficient support to prevent EMS activations for cardiac arrest patients with DNAR orders. Apart from home care, there is a pressing necessity to enhance the palliative care system. Challenges include improving palliative care for non‐cancer patients,^45^ and integrating palliative care with other specialties, such as intensive care units, thereby enhancing the likelihood of patients receiving palliative care or engaging a palliative care team in goals‐of‐care discussions.^46^ These proposed measures are potential solutions to reduce unwanted CPR in patients with DNAR orders.

### Limitations of this study

First, our search was limited to English and Japanese publications. Nevertheless, this approach seemed appropriate given the topic's connection to Japan, where Japanese and English are the primary languages for such articles. Additionally, our search was confined to specific databases. It is important to note that the database for Japanese articles is renowned for its comprehensiveness, providing access to the most relevant publications. Second, the qualitative nature of our research aimed to encompass a wide range of viewpoints to offer a holistic perspective, but inherent bias is inevitable in qualitative studies. Instead of excluding studies based on bias assessment, we focused on including available studies and developing themes. These emerging themes accurately reflect the experiences and perspectives of authors, all current or former clinicians in Japan, and the broader discourse in the field. Third, as this review is based on existing literature identified through our search criteria, it is important to acknowledge that not all factors contributing to unwanted CPR may have been captured. For instance, social and psychological aspects that patients and family members may have concerning DNAR orders were not extensively covered. Given the complexity and multifaceted nature of this topic, it is conceivable that existing research may not encompass all dimensions. Our review should be viewed as a starting point for further exploration into this crucial area.

## Conclusion

This study identified the potential triggers for unwanted CPR despite DNAR orders in Japan. The multifaceted factors contributing to these instances include the lack of consensus on DNAR order format, communication deficits regarding ACP, the necessity for ethical education among healthcare professionals, legal climate and regulatory ambiguities, and challenges within the healthcare system. These findings highlight the pressing need for cohesive interventions encompassing education, ethical considerations, systemic changes, and legal modifications. By addressing these facets, Japan's healthcare landscape can evolve toward a more patient‐centered, ethically sound, and regulatory coherent approach to aligning medical practices with patients' end‐of‐life preferences.

## Funding information

This research did not receive any specific grant from funding agencies in the public, commercial, or not‐for‐profit sectors.

## Disclosure statement

The authors declare no conflict of interest regarding this article.

## Author contributions

KK: conceptualization, methodology, formal analysis, investigation, visualization, writing – original draft, project administration. KI: conceptualization, methodology, writing – review and editing. Takeshi Uemura: conceptualization, methodology, formal analysis, visualization, writing – review and editing, supervision.

## Ethics statement

Not applicable.

## Patient consent statement

Not applicable.

## Clinical trial registration

Not applicable.

## Supporting information


**Table S1.** Search terms for literature review.
**Table S2.** Inclusion and exclusion criteria of the literature review.
**Table S3.** Summarized findings of the included studies.

## Data Availability

The data that support the findings of this study are available from the corresponding author upon reasonable request.
