# Supplementary material for: Unwanted cardiopulmonary resuscitation against patients’ “Do Not Attempt Resuscitation” orders in community settings in Japan: A narrative review
Source: Geriatr Gerontol Int. 2024 Oct 1;24(11):1093–8. doi: 10.1111/ggi.14993 (PMC11843528; doi:10.1111/ggi.14993)
Supplement: Supplementary file 1 — Table S1. Search terms for literature review. Table S2. Inclusion and exclusion criteria of the literature review. Table S3. Summarized findings of the included studies. [file GGI-24-1093-s001.docx]

**APPENDIX A. Supplementary material**

**Table 1. Search terms for literature review**

| **Search engine** | **Search terms** |
| --- | --- |
| PubMed (in English) | (Cardiopulmonary resuscitation) AND (resuscitation orders) AND (Japan)  (life support care) AND (resuscitation orders) AND (Japan)  (resuscitation orders) AND (Japan) |
| EMBASE (in English) | (resuscitation) AND (do not resuscitate order) AND (Japan)  (life sustaining treatment) AND (do not resuscitate order) AND (Japan)  (do not resuscitate orders) AND (Japan) |
| Scopus (in English) | (cardiopulmonary resuscitation) AND (do not resuscitate order) AND (Japan)  (life sustaining treatment) AND (do not resuscitate order) AND (Japan)  (do not resuscitate orders) AND (Japan) |
| Google Scholar (in Japanese) | (Cardiopulmonary resuscitation (shinpai-sosei)) AND (DNAR)  (Cardiopulmonary resuscitation (shinpai-sosei)) AND (DNR) |

**Table 2. Inclusion and exclusion criteria of this literature review**

| Inclusion criteria | Exclusion criteria |
| --- | --- |
| -published in either English or Japanese  -published by 12/2022  -both peer-reviewed articles and gray literature  -both quantitative and qualitative studies  -complete full-text articles are available  -articles relevant to our core research question: "What are the underlying reasons for unwanted CPR despite DNAR preferences in community settings in Japan?" | -not written in English or Japanese  -any review articles  -case reports and case series  -articles that focused solely on resuscitation procedures without addressing patient wishes or preferences for life-sustaining interventions |

**Table 3. Summarized findings of the included studies**

| **Reference** | **Study aim** | **Date of data collection** | **Sample** | **Method** | **Main Results** |
| --- | --- | --- | --- | --- | --- |
| Maruhashi T, et al, 2021. [^7^](https://paperpile.com/c/SMUzm4/agQvu) | To investigate the utilization of DNAR directives and EMS interventions in instances of OHCA within Japan | Between May 30, 2019, and February 15, 2020 | All cardiac arrest cases in Sagamihara Municipal Fire Department EMS team | Cross-sectional study | Among 396 instances of OHCA, a significant majority (80.6%) of cases pertained to individuals aged 65 or older.  Forty-five cases (11.4%) were associated with confirmed DNAR orders, of which 12 cases (26.7%) were documented in written form.  CPR procedures were executed in 43 cases (95.6%) of the total cohort. |
| Tanabe R, et al, 2022. [^8^](https://paperpile.com/c/SMUzm4/0Yrrm) | To elucidate the attributes and consequences of patients who experience OHCA while concurrently discerning the variables linked with heightened stress levels among EMTs during the provision of medical care to OHCA patients subject to DNAR directives | Between 2015 and 2019 | 3079 OHCA patients for descriptive analysis, and 243 EMTs for a survey in Okayama city | Cross-sectional study | In the case-cohort comprising 3,079 OHCA patients, it was observed that 122 individuals (approximately 4%) had been issued DNAR directives.  Among this subgroup of 122 patients with DNAR directives, 120 (98%) underwent CPR. Meanwhile, within 2,957 patients without DNAR orders, 2,915 patients (99%) received CPR.  Within 243 EMTs involved, approximately 30% of EMTs reported experiencing elevated stress levels when administering CPR to OHCA patients with DNAR directives. This stress stems from the conflict between the professional obligation to perform CPR and the presence of a DNAR order.  Only 9 EMTs (4%) confirmed the existence of written documentation for patients with DNAR orders. In contrast, 94 out of 173 EMTs (54%) indicated that they discerned the presence of DNAR directives either through verbal communication or written documentation before initiating CPR.  155 EMTs (65%) reported implementing what is commonly referred to as "slow code" practices.  The frequency of administering CPR to patients with DNAR orders exhibited a noteworthy difference when compared to analogous situations in the U.S., the United Kingdom, and France, showcasing a notably higher occurrence. |
| Mayumi T, et al, 2017.[^9^](https://paperpile.com/c/SMUzm4/cGa7P) | To investigate characteristics of cardiac arrest patients with frailty who were transported to the hospital | Between April 2010 and March 2012 | 43 cases whose clinical frailty scale is greater than seven who were transported to Ichinomiya municipal hospital due to cardiac arrest | Cross-sectional study | Out of the 43 cases, 15 patients had terminal cancer, seven patients were afflicted with neurodegenerative diseases such as ALS, and 21 patients were grappling with diminished ADL attributed to stroke or the aging process.  In 21 instances, EMS was summoned from nursing facilities or through home visiting nurses. In the remaining 22 cases, EMS were called from private residences.  Among the total cases, 16 instances (37.2%) involved the presence of DNAR orders. Intriguingly, in 8 cases, EMS was contacted by personnel from nursing homes or home-visiting nurses, none of whom were aware of the patients' DNAR preferences. In the same number of cases, EMS were called from private homes. Among these 8 cases, in 5 situations, the patient's family informed EMS about the patient's DNAR wishes; however, CPR procedures were still administered. |
| Tokuda Y, et al, 2004.[^10^](https://paperpile.com/c/SMUzm4/R3Z4W) | To compare and assess decision-making for end-of-life care for patients with cancer at a tertiary teaching hospital in Japan at two periods ten years apart | In 1989 and 1999 | 63 patients with cancer were admitted to the hospital in 1989 and 61 patients with cancer who were admitted to the hospital in 1999. The hospital is a tertiary teaching hospital with 550 beds. | Cross-sectional study | The proportion of patients exhibiting cognitive competency was 76.2% and 80.3% in the respective periods.  None of the patients were informed of their cancer diagnosis in 1989, whereas five patients (8.2%) received such information in 1999.  Discussions surrounding DNR directives occurred with 56 patients (88.8%) in 1989 and 57 (93.4%) in 1999.  Although records documented conversations on DNR, there were no formally signed consent forms for patients and their families. DNR orders were endorsed by either residents or attending physicians before patients' demise.  CPR was administered to 15 patients, often at the insistence of their families. Six patients (3 in 1989 and 3 in 1999) underwent CPR based on family requests despite the existence of a DNR directive.  In both 1989 and 1999, patients were subjected to total parenteral nutrition (6 patients, 10% and eight patients, 13%, respectively), albumin infusion (4 patients, 6% and five patients, 8%, respectively), and tube feeding (5 patients, 8% and five patients, 8%, respectively). |
| Toma M, et al, 2020. [^11^](https://paperpile.com/c/SMUzm4/mb5El) | To report the status of pre-hospital DNAR orders in terminal cases in Japan | Between January 1, 2014, and December 31, 2018 | 5513 cardiac arrest cases in Harima Medical Control Council | Cross-sectional study | Out of a total of 5513 cardiac arrest cases, 270 cases (4.9%) were associated with DNAR preferences, with only 15 cases (5.6%) having documented written DNAR requests.  Among these cases, 156 instances (57.8%) necessitated EMS intervention from private residences, while 105 cases (38.9%) sought EMS assistance from elderly care facilities.  Of the total, 22 cases (8.1%) were not transported to hospitals due to the presence of PCP, whereas 248 cases (91.9%) were conveyed to medical institutions for further treatment.  Among the transported cases, 179 (72.2%) were taken to hospitals and received CPR without endotracheal intubation, whereas 67 cases were subjected to CPR and intubation.  A mere 29% of medical facilities possessed a collaborative system with other healthcare institutions to facilitate end-of-life care. |
| Hiraoka E, et al, 2016.[^12^](https://paperpile.com/c/SMUzm4/yVgbc) | To investigate how DNR orders affect decision-making on whether life-sustaining procedures, including non-CPR procedures, are performed | In 2016 (Details not written) | 161 physicians from 3 municipal acute care hospitals in Japan (111 physicians answered, response rate 69%) | Cross-sectional study (questionnairee using 3 case scenarios of advanced lung cancer, advanced dementia, and non-advanced heart failure) | In all three scenarios, there was a noticeable reduction in the proportion of physicians willing to undertake non-CPR procedures when faced with DNR orders. The median percentages and interquartile ranges were as follows: Case 1: 72% [45%–90%] without DNR orders versus 100% [90%–100%] with DNR orders; Case 2: 55% [36%–72%] without DNR orders versus 91% [63%–100%] with DNR orders; Case 3: 78% [55%–88%] without DNR orders versus 100% [88%–100%] with DNR orders.  Many physicians indicated their willingness to perform electric cardioversion for ventricular fibrillation even when DNR orders were in place (Case 1: 26%; Case 2: 16%; Case 3: 20%). The authors concluded that there is a pressing need for precise definitions of "Cardiac arrest" and DNR to be established and clarified within the context of the medical landscape in Japan. |
| Ethics Committee, Japanese Society of Intensive Care Medicine, 2017. [^13^](https://paperpile.com/c/SMUzm4/P7uvl) | To investigate the clinical situation of DNAR implementation and recognition of DNAR orders by nurses | Between October 13 and October 27, 2016 | 384 out of 2352 nurses who are members of the Japanese Society of Intensive Care Medicine (16.3% response rate) | Cross-sectional study | Among the responses provided by nurses, the withholding of circulatory assist devices like PCPS and ECMO was the most frequent procedure when a DNAR order was in place. Following this, CPR was the second most withheld procedure for patients with DNAR orders.  The clinical situation nurses considered appropriate for implementing a DNAR order was primarily when a patient had terminal cancer, followed closely by a clear expression of the patient's DNAR preferences.  Regarding decision-making involvement, 16 nurses (6.5%) indicated that patients are consistently included in DNAR decisions, while 104 nurses (42.1%) stated that patients are sometimes involved. In contrast, most 262 nurses (92.1%) affirmed that family members are always involved in DNAR decision-making.  A total of 85 nurses (28.2%) disclosed that their facilities have a manual or guidelines in place for the execution of DNAR orders. |
| Ethics Committee, Japanese Society of Intensive Care Medicine, 2017. [^14^](https://paperpile.com/c/SMUzm4/puTh) | To investigate understanding and implementation of DNAR orders among providers and its effects on non-CPR procedures | Between October 13, 2016, and October 27, 2016 | 86 out of 261 Society Counselors (response rate 33.0%), and 595 out of 6588 physicians in the Japanese Society of Intensive Care Medicine | Cross-sectional study | Among the 86 Society Counselors, 46 respondents expressed that CPR should be avoided in patients with DNAR orders. However, a significant number of responders suggested that other procedures, including dialysis, PCPS/ECMO, administration of pressors, intubations, and other invasive measures, should be withheld in patients with DNAR orders.  Of the 595 physicians surveyed, 44.4% stated that discussions about DNAR orders involve multiple medical staff members, including various physicians, the respondent, and other medical personnel. Furthermore, 28.2% of physicians indicated that DNAR orders are discussed among the respondents and other physicians, while 16.4% stated that they make DNAR decisions independently.  Regarding patient involvement in DNAR discussions, 215 physicians (39.8%) reported that they have engaged in DNAR conversations with the patient present, whereas 325 physicians (60.2%) revealed that they have never discussed the patient's DNAR order in the presence of the patient. |
| Sato T, et al, 2020.[^15^](https://paperpile.com/c/SMUzm4/h1YQV) | To assess how the Japanese version of “POLST preparation guideline” published in 2015 was utilized | Between December 2018 and March 2019 | Chairs of the Ethics Committee of a total of 636 hospitals nationwide (195 hospitals answered) | Cross-sectional study | Among the 195 hospitals surveyed, 84 hospitals (43%) possessed documented DNAR orders. Of these, a substantial majority, 82 hospitals (96%), exclusively utilized their forms rather than standardized shareable forms. In 169 out of 191 hospitals (88%), respondents acknowledged experiencing confusion and difficulties implementing DNAR orders.  Approximately 84 hospitals (44%) stated that they had never encountered a POLST form.  Most 104 hospitals (55%) indicated that they discontinued medical procedures other than CPR per DNAR orders.  Concerning discussions about the goals of care, 98 hospitals reported engaging in conversations with patients who possessed the capacity to make decisions. Meanwhile, 99 hospitals revealed that such discussions extended to the patient's family, even in cases where the patient had the capacity to decide. Interestingly, 26 hospitals reported conducting both types of discussions. |
| Fujimoto K, et al, 2014.[^16^](https://paperpile.com/c/SMUzm4/Roae7) | To compare the timing and decision makers of DNR orders between patients with end-stage thoracic cancer and non-cancer respiratory disease in acute care hospitals in Japan | between January 2008 and March 2013 | 300 cancer patients and 147 non-cancer patients who had DNAR orders and died in the study period, in the Department of Respiratory Medicine of Osaka Police Hospital (tertiary hospital) | Retrospective cohort study | Cancer patients exhibited a higher prevalence of deciding on a DNR order in advance before their last hospital admission compared to non-cancer patients (21.0% vs. 10.2%). Furthermore, cancer patients were more likely to possess normal cognitive function in contrast to non-cancer patients (61.7% vs 38.8%).  Within both groups, only approximately 6% of all patients participated in discussions concerning their own DNR preferences.  Even when considering a subset of 185 cancer patients and 57 non-cancer patients without cognitive impairment, a mere 14 individuals (7.6%) and 6 individuals (10.5%), respectively, were actively engaged in DNR discussions. |
| Kizawa Y, et al, 2013.[^17^](https://paperpile.com/c/SMUzm4/0Xcxn) | To examine the status of AD and DNAR orders among patients with terminal cancer in PCUs in Japan | Between December 2010 and February 2011 | 297 patients died in 203 PCUs across Japan before November 2010. | Cross-sectional study | Among the total of 297 patients, 140 individuals (47%) had AD in place specifically related to CPR, while 138 patients (46%) had AD on mechanical intubation. Furthermore, 143 patients (48%) had designated a healthcare proxy.  Among the subgroup of 214 patients who possessed the capacity to make medical decisions, 111 patients (68%) actively participated in the process of making DNAR decisions, while 45 patients (28%) were not actively involved in the decision-making process. |
| Nakagawa Y, et al, 2017. [^18^](https://paperpile.com/c/SMUzm4/lRmqW) | To investigate the status of DNAR orders and DNAR-related issues in Japan | Between August 15 and September 30, 2014 | 136 out of 292 hospitals in Kanagawa prefecture, Japan (hospitals specialized in obstetrics, pediatrics, and psychiatry were excluded) | Cross-sectional study | The practice of DNAR orders was observed in 88% of the surveyed hospitals, while 12% indicated that DNAR orders were not being implemented. Only 27% of hospitals had established in-house guidelines for DNAR.  A substantial 81% of hospitals reported that the proportion of patients involved in the decision-making process of their own DNAR order was limited to 25% or less (information regarding their decision-making capacity was not provided in the article).  When cardiac arrest occurred at home during clinic hours, the primary course of action taken for patients with DNAR orders was calling for an ambulance (58%). A secondary action involved a home visit by the primary care physician to pronounce death (27%). Similar trends were observed after clinic hours.  In instances where patients with DNAR orders experienced cardiac arrest at home, only 18% of institutions instructed EMS to perform CPR. A significant majority of physicians, approximately 70%, did not provide specific instructions and were left deciding whether to perform CPR on the EMS team. |
| Matsumoto T, et al, 2005. [^19^](https://paperpile.com/c/SMUzm4/WG0EG) | To clarify the situation of end-stage respiratory management utilizing DNAR sheet for patients with end-stage lung disease who were admitted to tertiary hospital | Between April 2003 to March 2004 | 75 patients who died of respiratory disease | Retrospective cohort study | The rate of acquiring a DNAR order was notably lower in patients with non-cancerous diseases in comparison to patients diagnosed with malignant diseases (33 out of 44 patients, or 75%, versus 31 out of 31 patients, or 100%). |
| Abe A, et al, 2021.[^20^](https://paperpile.com/c/SMUzm4/wzFSN) | To investigate patient participation rate in CPR/DNAR discussion and end-of-life (EOL) disclosure and its associated factors | Between April 2018 and March 2019 | 358 patients were hospitalized and died in Keio University Hospital (tertiary hospital). Cardiac arrest on arrival, stillbirth, underage 18, and refusal by their family were excluded. | Cross-sectional study | Discussions concerning CPR and DNAR were conducted with 336 out of 358 patients (93.7%).  Among these discussions, 224 took place without the patients' involvement, resulting in a patient participation rate of 33.3%.  Factors such as male gender, living alone, and one year or more since diagnosis were associated with higher patient participation rates in CPR/DNAR discussions.  EOL disclosure, which includes prognostic announcements, was carried out in 341 out of 358 patients (95.3%). Of these discussions, 170 occurred without patients, resulting in a patient participation rate of 50.3%.  Patients who passed away due to cancer and those without mental illness demonstrated a greater likelihood of participating in EOL disclosure discussions.  The observed participation rates were lower than those reported in previous studies conducted in the United States, Switzerland, and Finland. |
| Nemoto M, et al, 2019. [^21^](https://paperpile.com/c/SMUzm4/hXpfh) | To evaluate the effects of the new protocol to discontinue CPR when EMTs have PCP’s order on-site | Between December 1, 2017, and November 30, 2018 | 23 cases with CPR were applied to DNAR protocol, and 41 EMTs who used this new protocol | Cross-sectional study | In 23 instances, CPR was continued in 12 cases and discontinued in 11 cases. Out of 41 EMTs, 24 indicated that they experienced delays in obtaining orders from PCP, and 30 EMTs reported encountering difficulties on-site while attempting to make decisions. |
| Miyabayashi M, et al, 2019. [^22^](https://paperpile.com/c/SMUzm4/nfaaY) | To examine the emotions of EMTs who perform CPR for patients with terminal-stage cancer with DNAR order | January 2017 | 103 EMTs | Cross-sectional study | In 95.2% of cases, EMTs were informed by family members that the patient had a DNAR order while CPR was being administered.  Approximately 17.5% of cases had documented written DNAR orders, all originating from nursing facilities.  Most EMTs expressed the sentiment that, given the current legal framework, they felt obligated to adhere to the requirements of the Fire Service Act even if patients had DNAR orders.  Around half of the EMTs indicated they would have performed CPR even if the Fire Service Act did not exist. |
| Mayumi T, et al, 2017. [^23^](https://paperpile.com/c/SMUzm4/SgWp6) | To investigate the utilization of DNAR orders in elderly care facilities in Ichinomiya City, Japan | The survey was conducted in June 2012 | at 108 out of 181 elderly care facilities in Ichinomiya City (response rate 59.8%) | Cross-sectional study | Among the 108 facilities surveyed, 41% reported engaging in discussions about code status upon patient admission, while 44.8% did not discuss code status with patients. A mere 18.1% of the facilities possessed a manual for managing emergencies at home for their residents. Additionally, 43.6% of facilities indicated that they called for an ambulance in all cases of cardiac arrest, irrespective of the presence of DNAR preferences. |
| Suzuki Y, 2015. [^24^](https://paperpile.com/c/SMUzm4/SgWp6) | To investigate why terminal cancer patients with DNAR orders were required to request an ambulance | In 2014 | 19 paramedics | Qualitative study | The themes extracted in qualitative data analysis included “unestablished legally valid system regarding DNAR order,” “inadequate knowledge about ambulance operation,” “insufficient medical support for dying at home with DNAR order,” “lack of system regarding end of life care at the nursing home,” “overreliance on EMS in an emergency,” “family member’s wishes to avoid death at home,” and “panic and unpreparedness of the family member in an emergency.” |
| Fukuda T, 2011.[^25^](https://paperpile.com/c/SMUzm4/6JlZM) | To investigate the outcome of OHCA patients and their living will, including DNAR order | Between April 2009 and March 2010 | 304 OHCA patients who were transferred to St. Luke’s International Hospital’s Emergency and Critical Care Center (tertiary hospital) | Retrospective cohort study | Out of the 304 patients who experienced OHCA and underwent CPR, 126 patients were identified as having a living will, indicating their preferences for medical interventions. However, a living will was not established for the remaining 168 patients.  Among the 126 patients with documented living wills, a subset of 29 patients (23.0%) expressed their desire for DNAR measures. Among these patients, 8 exhibited ROSC and were admitted to the ICU, contrary to their previously stated DNAR preferences. One patient entered a comatose state as a result of these interventions. |
| Nishimoto Y, 2017.[^26^](https://paperpile.com/c/SMUzm4/6QNqR) | To clarify regarding instances where CPR is administered despite the absence of a desire for life-sustaining interventions | Between April 1st, 2015, and April 1st, 2016 | All patients who received CPR in 38 EMS stations in Hiroshima prefecture, Japan | Cross-sectional study | Twenty-three patients underwent CPR despite having either a living will specifying their wish to forgo CPR or due to the expressed intentions of their family members to discontinue CPR. Among these 23 patients, CPR was terminated in 20 cases when EMTs could reach out to PCP, who ordered the discontinuation of CPR. |
| Hosokawa K, et al, 2019. [^28^](https://paperpile.com/c/SMUzm4/LphcH) | To investigate how the protocol is implemented when EMS encounters cardiac arrest patients with DNAR wishes | Between April 2015 and March 2017 | 38 cardiac arrest cases whose DNAR wishes were found on site of CPR | Cross-sectional study | All 38 cases included in the study underwent CPR. In 1 patient, written documentation of DNAR wishes was discovered, while in the remaining 37 patients, DNAR wishes were verbally confirmed by family members or facility staff.  Among these cases, in 33 instances (87%), EMS successfully obtained orders from PCPs to halt CPR. In the remaining 5 cases (13%), ED physicians in hospitals or medical control issued orders to discontinue CPR.  Of the cases, 24 patients (63%) were transferred to a hospital, with CPR being continued for 10 (26%). For the remaining 14 patients (37%), hospital transfer did not occur. Among this group, CPR was sustained until a physician arrived at the patient's location in 4 cases (11%). *Patients whose DNAR wishes were ascertained after arriving at the hospital were excluded from this study. |
| Hosokawa K, et al, 2020. [^29^](https://paperpile.com/c/SMUzm4/KiT3k) | To assess the protocol regarding performing CPR for patients with DNAR order (EMTs must perform CPR until they can reach out to PCP to have an order to discontinue CPR) | December 2017 | 1079 staff who have worked as EMTs in Hiroshima Emergency Bureau | Cross-sectional study | Of the participants surveyed, 27% reported that they had encountered instances where CPR was performed on patients with DNAR orders. Among this subset, 38% of respondents indicated that they faced difficulties in obtaining approval from PCP to cease CPR while they were in the process of administering it. |
| Yaegashi M, et al, 2021. [^30^](https://paperpile.com/c/SMUzm4/41IBl) | To evaluate the effectiveness of a novel system designed to accommodate end-of-life terminal patients' preferences to remain at home while ensuring that they can be transported to the hospital without undergoing CPR and to facilitate the seamless communication of these patient wishes between ambulance staff and family members | Between November 2018 and January 2020 | A total of 37 ​​ terminal patients who wished to spend their last moments at home | Prospective cohort study | Out of the 37 terminal patients included in the study, a total of 26 patients passed away during the designated study period. Among these, eight patients were transferred to a hospital to have their deaths officially pronounced by a medical doctor, and CPR was not performed for any of these patients. |

*Abbreviations: DNAR: Do Not Attempt Resuscitate; EMS: Emergency Medical Service; OHCA: Out-of-Hospital Cardiac Arrest; CPR: Cardiopulmonary Resuscitation; EMT: Emergency Medical Technician; the U.S.: the United States; ROSC: Return of Spontaneous Circulation; ICU: Intensive Care Unit; ALS: Amyotrophic Lateral Sclerosis; ADL: Activities of Daily Living; DNR: Do Not Resuscitate; PCP: Primary Care Physician; POLST: Physician Order for Life-Sustaining Treatment; PCPS: Percutaneous cardiopulmonary support system; ECMO: Extracorporeal membrane oxygenation; AD; Advance Directive; PCU; Palliative Care Units, EOL; End-of-life.
